# Supplementary material for: Metabolic dysfunction-associated steatotic liver disease (MASLD) can be a possible predictive factor of incident CKD: NAGALA cohort study
Source: PLoS One. 2025 Oct 17;20(10):e0334720. doi: 10.1371/journal.pone.0334720 (PMC12533907; doi:10.1371/journal.pone.0334720)
Supplement: Attachment — Submitted filename: Review of Article-20250512.docx [file pone.0334720.s001.docx]

**Summary:**

The author conducted a retrospective cohort study to investigate whether metabolic dysfunction-associated steatotic liver disease (MASLD), a recent redefinition of metabolic dysfunction-associated fatty liver disease (MAFLD), independently predicts chronic kidney disease (CKD). Data from 15,873 individuals attending health checkups from 1994–2023 at Asahi University Hospital in Japan were analyzed. Over five years, CKD incidence was highest among participants with MASLD (9.5%). Logistic regression analysis showed MASLD significantly increased CKD risk (OR 1.37; 95% CI, 1.12–1.67; p = 0.002). Age (OR 1.04; p < 0.001) and baseline estimated glomerular filtration rate (OR 0.88; p < 0.001) were also notable risk factors. Results confirmed that MASLD independently elevates CKD risk beyond traditional cardiometabolic risk factors. Thus, the author provided a novel insight that early detection and targeted management of MASLD could substantially reduce CKD incidence. However, there are still some comments need to be addressed by the author.

**Comments:**

1. In Introduction part, please give clearer definition for “MetALD" and "cryptogenic SLD" to avoid confusion.
2. The phrase "contemporary clinical priorities" (line 67) is vague; specifying these priorities (e.g., metabolic health, cardiovascular risk) could improve clarity.
3. You mentioned the study began in 1994 (lines 84–86), but participants were included from 2004 to 2023 (lines 88–89). Clearly explain the discrepancy or revise for consistency.
4. Why did Groups 3 and 4 both labeled "A- or +/C-/S+"? I speculate each group should have a unique and clearly differentiated label.
5. Clarify whether "A-, + or +++" indicates optional alcohol consumption or limited alcohol intake, and ensures consistency across groups.
6. The criteria for excluding participants with “existing liver disease” or “history of medication use” are overly broad. Specify medication categories or clarify liver disease criteria to prevent ambiguity.
7. Clearly distinguish definitions, since “non-alcohol consumers” (<210g/week men, <140g/week women) still implies some alcohol intake. Consider re-labeling categories as "minimal," "light," and "moderate-heavy" drinkers for clarity.
8. Please correct the inconsistent sample sizes for Group 1 and Group 2: Text (line 161): Group 1 = 6,216; Group 2 = 7,568, but Table 2 excerpt: Group 1 = 5,784.
9. Also, please adjust the format of content, only half of Table 2 was presented in the manuscript.
10. The model adjusts for age, sex, eGFR, exercise, and smoking status—but does not include BMI, blood pressure, or lipids, which are relevant confounders in CKD development.
11. In tables and text, use consistent terminology: “Fasting prandial glucose” should be either “fasting glucose” or “plasma glucose.” Please use "CKD incidence" rather than "CKD rate" when referring to outcomes in a statistical context.
