# Supplementary material for: Metabolic dysfunction-associated steatotic liver disease (MASLD) can be a possible predictive factor of incident CKD: NAGALA cohort study
Source: PLoS One. 2025 Oct 17;20(10):e0334720. doi: 10.1371/journal.pone.0334720 (PMC12533907; doi:10.1371/journal.pone.0334720)
Supplement: Attachment — Submitted filename: Rebuttal_Letter_CKD_MASLD_updated.docx [file pone.0334720.s002.docx]

Title of Manuscript: Metabolic dysfunction-associated steatotic liver disease (MASLD) can be a possible predictive factor of incident CKD: NAGALA cohort study

Dear Reviewer,

We sincerely thank you for your valuable and insightful comments. We have carefully addressed each of your points, as detailed below. All changes have been incorporated into the revised manuscript.

**Reviewer #1:**

This manuscript presents valuable findings on MASLD as a risk factor for CKD, but revisions are needed to address inconsistencies in group sizes, clarify non-significant associations, and account for low-powered subgroup analyses.

**Response:** Thank you for your valuable comments. We carefully reviewed the group sizes and confirmed that there were no discrepancies between the text and tables. Regarding non-significant associations, we clarified in both the Results and Discussion sections which findings were not statistically significant and interpreted them with appropriate caution. As for subgroup analyses, we recognize that the statistical power was limited, particularly in small groups such as Group 4 and Group 5. These groups consisted of only 2 and 4 participants, respectively, and no incident CKD cases occurred during follow-up. We have emphasized this limitation in the revised Discussion and clearly stated that no conclusions can be drawn regarding CKD risk in these small subgroups. We believe this careful interpretation helps preserve the integrity of our findings and avoids overstatement of results.

**Reviewer #2:**

This study presents valuable insights into the relationship between MASLD and CKD. The research design is robust, and the findings are clinically relevant. However, the study’s retrospective nature and reliance on a single hospital population may limit generalizability. Also, mechanisms were not explored.

**Response:** We sincerely appreciate your positive evaluation of our research design and findings. We have explicitly noted the retrospective design and single-center limitation in the Discussion section, and emphasized that further multicenter prospective studies are needed to enhance generalizability. We also included a new paragraph in the Discussion to suggest future research directions focusing on the biological mechanisms linking MASLD to CKD, including inflammation, oxidative stress, and insulin resistance.

**Reviewer #3:**

I have only a few suggestions. In recent years, there has been increasing interest in the concept of cholemic nephropathy and early renal function impairment in patients with mild hyperbilirubinemia. Consider discussing this with reference to DOI: 10.1111/liv.16005

**Response:** Thank you for this insightful suggestion. We have now added a discussion on the potential impact of acute hyperbilirubinemia and the emerging concept of cholemic nephropathy in the revised Discussion section. The recommended article (DOI: 10.1111/liv.16005) has been cited to provide appropriate context as follows.

Van Slambrouck CM, Salem F, Meehan SM, Chang A. Bile cast nephropathy is a common pathologic finding for kidney injury associated with severe liver dysfunction. Liver Int. 2013 Apr;33(4):512–20.

DOI: 10.1111/liv.16005 ↩

**Reviewer #4:**

1. The paper would have been more complete if it had compared the prevalence or incidence using both the old and new nomenclature.
2. More emphasis should be placed on analysis between groups. Hazard ratio terminology may be inappropriate.
3. The role of medications in CKD onset/prevention is not addressed. Competitive risk analysis would be more suitable.
4. CKD onset timing is not available.

**Response:**

1. Thank you. We appreciate the suggestion to compare the previous (MAFLD/NAFLD) and current (MASLD) nomenclature. However, as our primary focus was on the new classification, and due to retrospective data limitations, we were unable to robustly apply the prior definitions. We have acknowledged this as a limitation and a point for future study.
2. We agree that clarification is needed. We have replaced "hazard ratio" terminology with the correct use of "odds ratio."
3. We appreciate this important comment. Due to lack of comprehensive medication data (e.g., RAS inhibitors, SGLT2 inhibitors), we could not assess drug effects on CKD incidence. We have acknowledged this limitation explicitly in the Discussion.

Furthermore, medication use was not systematically recorded in our dataset. Therefore, we were unable to assess the potential influence of renin–angiotensin system (RAS) inhibitors, SGLT2 inhibitors, or other nephroprotective or nephrotoxic agents on CKD incidence. This represents a limitation, as these drugs may significantly modify renal outcomes and confound associations between MASLD and CKD.

4. The specific timing of CKD onset during the 5-year follow-up was not available in our dataset. This limitation has now been clearly mentioned in the revised manuscript.

In addition, due to the nature of the health checkup dataset, the exact timing of CKD onset during the 5-year follow-up period could not be determined. This prevented us from performing time-to-event analyses such as Cox proportional hazards or competing risk models.

**Reviewer #5:**

1. Clarification of “MetALD” and “cryptogenic SLD”

Thank you for the suggestion. We have revised the sentence (lines 74–77) to explicitly define the terms: ‘cryptogenic SLD’ is defined as hepatic steatosis without cardiometabolic risk, irrespective of alcohol consumption, and ‘MetALD’ refers to MASLD with increased alcohol intake.

2. Clarification of “contemporary clinical priorities”

We agree. The phrase has been revised to specify “cardiometabolic risks” as an example of contemporary priorities (line 67–68).

3. Clarification of study period (1994 vs 2004–2023)

We clarified that although the NAGALA cohort was initiated in 1994, the present analysis included data from participants enrolled between 2004 and 2023 due to the availability of complete baseline information. In particular, waist circumference, which is essential to defining cardiometabolic risk, was only measured beginning in 2004. Therefore, we restricted our study period to 2004–2023 to ensure consistency and data completeness.

4. Duplicated group definitions (Groups 3 and 4)

We clarified in the text that although alcohol status overlaps (A- or +), Group 3 includes steatosis of unknown origin (“cryptogenic”), whereas Group 4 includes specific causes (e.g., drug-, virus-induced SLD).

5. Clarify alcohol categories: A-, A+, A+++

We revised the terminology as follows:
- A−: Minimal drinkers (<210 g/week men, <140 g/week women)
- A+: Light drinkers (210–420 g/week men, 140–350 g/week women)
- A+++: Moderate-heavy drinkers (>420 g/week men, >350 g/week women)

6. Clarify exclusion criteria: “existing liver disease” and “medication”

We revised the Methods section to specify that “existing liver disease” refers to viral hepatitis, autoimmune liver disease, and alcoholic hepatitis, and “medications” refer to hepatotoxic drugs such as corticosteroids and methotrexate. Importantly, these criteria are consistent with our previous study (Sakai et al., J Diabetes Investig. 2024;15:1788–1796), which adopted identical exclusion criteria within the same NAGALA cohort framework. This alignment ensures comparability and methodological consistency across our studies.

7. Alcohol intake definitions: 'non-alcohol consumers' terminology

We revised the categories to “minimal,” “light,” and “moderate-heavy” drinkers for clarity and consistency.

8. Inconsistent sample sizes in text vs Table 2

We corrected the sample size of Group 1 in the main text to match Table 2.

9. Incomplete presentation of Table 2

The full content of Table 2 has now been included and formatted appropriately in the revised manuscript.

10. Model adjustment variables: Missing confounders

Although we did not include BMI, blood pressure, or triglycerides in the main regression model to avoid multicollinearity—since these are part of the MASLD diagnostic criteria—we tested an alternative model including these variables. As expected, adjusting for them attenuated the differences in CKD incidence across the MASLD subgroups. This finding highlights the conceptual overlap between MASLD components and CKD risk factors. Therefore, we believe that excluding these variables from the main model was appropriate to avoid overadjustment bias. The results of the alternative models are provided in the Supplementary Materials.

11. Terminology consistency

We revised the terminology throughout the manuscript to ensure consistency (e.g., “fasting glucose,” “CKD incidence”).

We believe these revisions strengthen the manuscript and enhance its clarity and scientific rigor. We appreciate your thorough review and helpful suggestions.

Sincerely,

Masahide Hamaguchi, MD, PhD
On behalf of all authors
